# Fluorinated hybrid solid-electrolyte-interphase for dendrite-free lithium deposition

**DOI:** 10.1038/s41467-019-13774-2

**Published:** 2020-01-03

**Authors:** Rajesh Pathak, Ke Chen, Ashim Gurung, Khan Mamun Reza, Behzad Bahrami, Jyotshna Pokharel, Abiral Baniya, Wei He, Fan Wu, Yue Zhou, Kang Xu, Qiquan (Quinn) Qiao

**Affiliations:** 1 Department of Electrical Engineering and Computer Science, Center for Advanced Photovoltaics and Sustainable Energy, South Dakota State University, Brookings, SD 57007 USA; 2 School of Science and Key Lab of Optoelectronic Materials and Devices, Huzhou University, Huzhou, People’s Republic of China; 3 Electrochemistry Branch, Sensor and Electron Devices Directorate, Power and Energy Division, U.S. Army Research Laboratory, Adelphi, MD 20783 USA

**Keywords:** Energy science and technology, Materials science

## Abstract

Lithium metal anodes have attracted extensive attention owing to their high theoretical specific capacity. However, the notorious reactivity of lithium prevents their practical applications, as evidenced by the undesired lithium dendrite growth and unstable solid electrolyte interphase formation. Here, we develop a facile, cost-effective and one-step approach to create an artificial lithium metal/electrolyte interphase by treating the lithium anode with a tin-containing electrolyte. As a result, an artificial solid electrolyte interphase composed of lithium fluoride, tin, and the tin-lithium alloy is formed, which not only ensures fast lithium-ion diffusion and suppresses lithium dendrite growth but also brings a synergistic effect of storing lithium via a reversible tin-lithium alloy formation and enabling lithium plating underneath it. With such an artificial solid electrolyte interphase, lithium symmetrical cells show outstanding plating/stripping cycles, and the full cell exhibits remarkably better cycling stability and capacity retention as well as capacity utilization at high rates compared to bare lithium.

## Abstract (web summary)

Here the authors report a simple method to create a solid electrolyte interphase that is tightly anchored onto the surface of lithium metal anode. This artificial structure suppresses dead and dendrite Li and stores Li via formation of alloys, enabling impressive battery performance.

## Introduction

Next-generation batteries based on lithium (Li) metal anodes, such as Li-air and Li-sulfur have been extensively studied owing to the high theoretical capacity (3860 mAh g^−1^, low density (0.59 g cm^−3^), and low redox potential (−3.04 V versus standard hydrogen potential) of Li^1^. Li batteries need to possess a higher capacity in order to integrate with renewable energy sources^2–4^. However, nonuniform Li plating, the infinite volume change of Li during plating/stripping, and the formation of fragile solid-electrolyte-interphase (SEI) lead to the growth of Li dendrites and formation of “dead Li”. Such irreversibility consumes both Li and electrolyte, leading to sustained capacity fading and low coulombic efficiency (CE)^5^. Li dendrites and “dead Li” also cause severe safety concerns of any batteries based on Li metal anode^6^. Substantial efforts have been done to deposit Li metal in a dense and reversible manner. For example, conventional copper foils have been replaced by advanced current collectors with nanostructured morphology to lower the current density and manipulate Li deposition sites^7^. Modifications of the separator have also been performed^8,9^. The use of solid or gel electrolytes also promises various advantages over volatile and flammable organic liquid electrolytes in their potentials to prevent parasitic reactions with Li, while providing excellent flexibility^10–12^.

Engineering ex situ protective layers have attracted much attention for Li metal batteries (LMBs). Improving the modulus properties and ionic conductivity of the interphase by various strategies have been reported^13,14^. The poor Li contact between these interfacial layers and bulk Li could lead to an increase in both interfacial and overall cell resistance. The low wettability of interphase towards nonaqueous electrolyte also leads to sluggish Li-ion transport. Differing from surface engineering of the artificial interphase layer, the use of various electrolyte additives^7,15^ provides an alternative pathway, where a more intimate contact could be ensured.

Recently, fluorinating SEI with LiF as a key component has been widely adopted to improve the cycling performance of Li metal anode based on two hypotheses: (1) LiF is an excellent electronic insulator whose wide gap effectively prevents electron tunneling^16^; (2) When interfacing with other ingredients at nanoscale, LiF could provide a high ionic conductivity, low diffusing energy, and high surface energy, which not only allows sufficiently fast Li-ion kinetics but more importantly promotes the electrodeposition of Li in a parallel rather than vertical manner^17,18^. Consequently, LiF-based interphase ensures better surface morphology and serves as a robust barrier to Li dendrite growth^17–24^. Besides LiF, Li-based alloys have also been studied as protective interphase to suppress Li dendritic growth, because Sn–Li alloy phase could reduce the Li-ion diffusion barrier, and lead to improved Li metal interphase stability^25–27^. Such alloy approaches include the in situ formation of Sn–Li, Li_13_–In_3_, Li–Zn, Li_3_–Bi, Li_3_–As, Au–Li, Si–Li, etc^25,28^. However, the development of an artificial SEI is still at its early stages. The mechanical and electrochemical instability of interphase leads to persistent deterioration. Low Li-ion conductivity, chemical instability, morphological inhomogeneity, and the subsequent uneven growth of natural SEI remain to be unresolved. In particular, there has never been synergy established between inert but protective LiF, the electrochemically active Sn and Sn–Li alloy on the SEI.

With these considerations in mind, we report a one-step approach to create an artificial SEI composed of LiF, Sn, and Sn–Li alloy tightly anchored to the Li surface. The artificially generated hybrid SEI not only eliminates Li dendrite and dead Li, but simultaneously stores Li via the formation of an alloy and enables Li plating underneath it. The hybrid SEI-modified Li symmetrical cells show outstanding plating/stripping cycles (~2325 h) with reduced overpotential compared to the bare Li. When coupled with a high-loading (11.88 mg cm^−2^) LiNi_1/3_Co_1/3_Mn_1/3_O_2_ cathode, Li full cells exhibit remarkably higher performance than bare Li anode in cycling stability, capacity retention, and capacity utilization at higher rates. To the best of our knowledge, this is the first demonstration of hybrid SEI that provides new solutions to the challenges in Li metal anodes.

## Results

### Preparation of an artificial fluorinated hybrid SEI

**Fig. 1:**
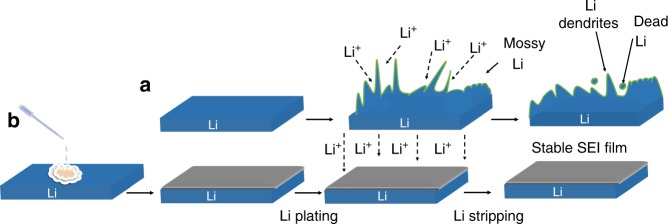
Schematic illustration of Li dendrites growth on bare Li and smooth Li deposition on artificial SEI-protected Li.

Figure 1a shows the growth of Li dendrites and “dead Li” on the bare Li metal after plating and stripping cycles. Figure 1b exhibits the fabrication process of fluorinated hybrid SEI by treating Li with SnF_2_ to obtain dendrite-free Li plating/stripping. By casting an electrolyte containing SnF_2_ on the surface of the Li metal electrode, a replacement reaction between Li metal and SnF_2_, and an alloying reaction between Li metal and Sn occur as follows^20,25^.

1 $${\mathrm{SnF}}_2 + 2{\mathrm{Li}} \to 2{\mathrm{LiF}} + {\mathrm{Sn}}$$

2 $$5{\mathrm{Li}} + 2{\mathrm{Sn}} \leftrightarrow {\mathrm{Li}}_5{\mathrm{Sn}}_2$$

**Fig. 2:**
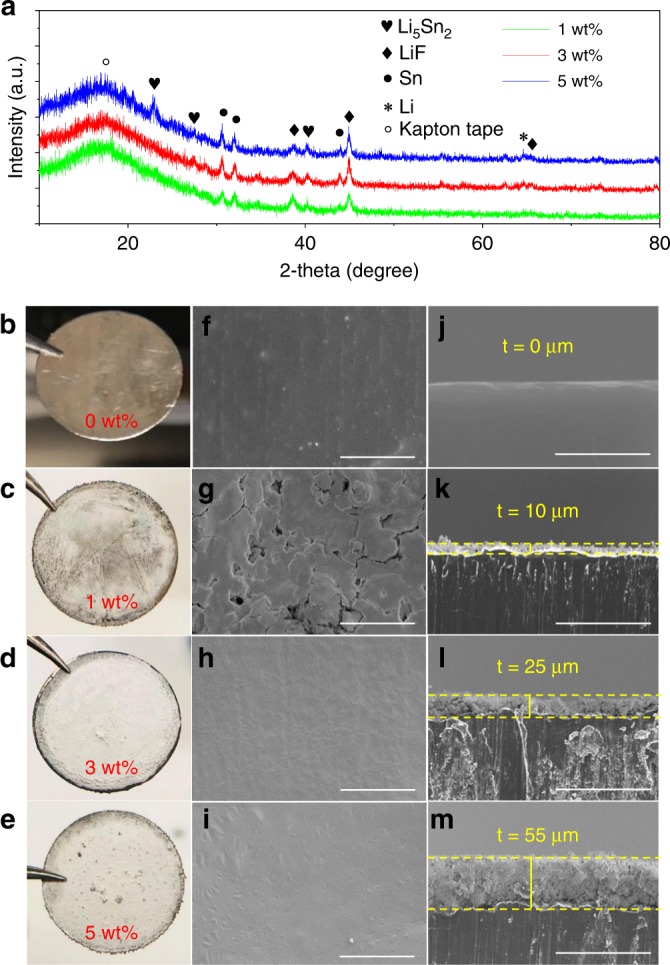
Structural and morphological characterizations. **a** XRD spectrum of Li electrode treated with 1, 3, and 5 wt% SnF_2_. **b**–**e** Photographic images of Li with different weight percentage concentrations of SnF_2_. **f**–**i** The corresponding top-view SEM images from **b** to **e**. The scale bars are 20 μm. **j**–**m** The corresponding cross-sectional SEM images from **f** to **i**. The scale bars are 100 μm.

Electrolytes with different concentrations of SnF_2_ (1 wt%, 3 wt%, and 5 wt%) were cast on bare Li electrodes. X-ray diffraction (XRD) measurements were performed to characterize the phase change of Li and investigate the components of the artificial SEI layer. To avoid the direct contact of the electrode with air or moisture, all the samples were measured under the protection of Kapton tape that has a wide diffraction peak at ~20°. Figure 2a shows the XRD spectra of artificial SEI layers generated on the Li electrode by treating with electrolytes containing 1, 3, and 5 wt% of SnF_2_. The pure Li metal shows the XRD peaks at ~36°, 52°, and 65° as shown in Supplementary Fig. 1. The artificial SEI layer constitutes a beneficial Sn–Li alloy (Li_5_Sn_2_), LiF, and Sn. Peaks at ~31.1°, 32.4°, and 44.1° correspond to Sn. Peaks at ~23.4°, 27.5°, 40.5° correspond to Li_5_Sn_2_. Peaks at ~38.7°, 44.9°, and 65.4° correspond to LiF^24,25,29^. With increasing concentration of SnF_2_, the peak strength of LiF and Li_5_Sn_2_ increases, and a new peak at ~23° appears that corresponds to Li_5_Sn_2_^25,30^. As the artificial fluorinated hybrid SEI formed on top of Li has a thickness of 10 µm or higher, this might be the reason for the minimal Li peak at 65°. As Li_5_Sn_2_, Sn, and LiF are on top of the Li electrode, this is why their peaks are pretty strong.

The silver shiny surface of bare Li metal (Fig. 2b) appears dark grey, immediately after treatment and turns to whitish when fully dried (Fig. 2c–e). Figure 2c shows that with 1 wt% SnF_2_, the artificial SEI does not completely cover or protect the Li surface. With 3 and 5 wt% SnF_2_ treatment (Fig. 2d, e), full coverage of the artificial SEI layer can be observed. The scanning electron microscope (SEM) image of the bare Li (0 wt% SnF_2_, Fig. 2f) shows a rough surface. Figure 2g–i shows the topography SEM image of the artificial SEI layers obtained using an electrolyte containing 1, 3, and 5 wt% SnF_2_, respectively. The SEI layer with 1 wt% SnF_2_ has pinholes (Fig. 2g) on the surface that allow penetration of electrolyte, resulting in the side reactions with the Li underneath. As a result, the electrolyte and Li are consumed that leads to low CE and capacity decay^31^. As a comparison, SEM images of the artificial SEI layers with 3 and 5 wt% SnF_2_ (Fig. 2h, i) do not show any pinholes or cracks that avoids direct contact of electrolyte with the Li underneath. The cross-sectional SEM image (Fig. 2j–m) shows that the average thickness (t) of the artificial SEI treated with an electrolyte containing 1, 3, and 5 wt% SnF_2_ is 10 µm, 25 µm, and 55 µm, respectively. The thicker SEI has a higher Li-ion barrier energy or higher impedance resulting in slow Li-ion diffusion^32^. Thus, the SEI layer thickness should be optimized in order to protect Li physically to avoid direct contact with the electrolyte. This will lead to high Li-ion conductivity. The Li protected by an artificial fluorinated hybrid SEI with a thickness of 10 µm, 25 µm, and 55 µm is abbreviated as AFH-10, AFH-25, and AFH-55, respectively.

### Li plating/stripping performance and impedance measurement of symmetric cells

**Fig. 3:**
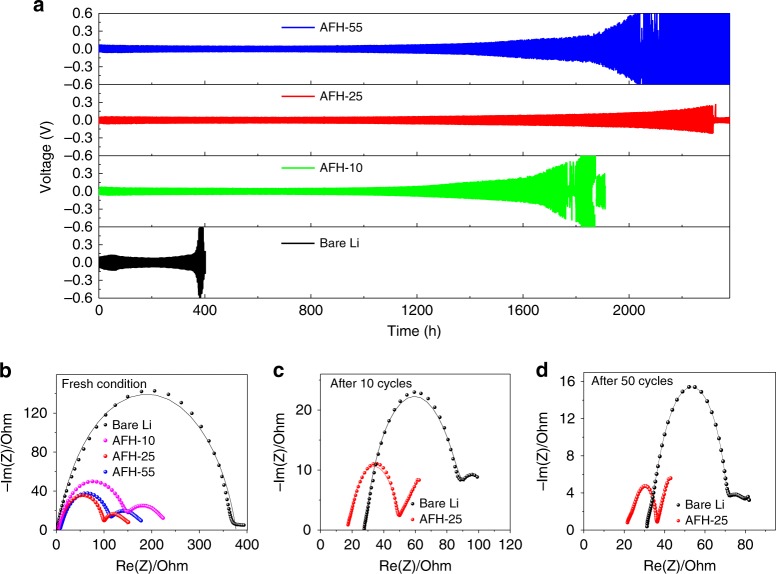
Electrochemical symmetrical cell test and EIS measurement. **a** The voltage profiles of symmetrical cells with bare Li and different thickness of AFH SEI at a current density of 0.5 mA cm^−2^ to achieve a capacity of 1 mAh cm^−2^. **b** Nyquist plot of bare Li and different thickness of AFH SEI symmetrical cells at fresh conditions. **c**, **d** Nyquist plot of bare Li and AFH-25 symmetrical cells after 10 cycles and 50 cycles at 0.5 mA cm^−2^, respectively.

Li plating/stripping tests were carried out to characterize the SEI layers. Figure 3a shows the comparison of the voltage–time profile of Li symmetric cells with different thicknesses of an artificial SEI generated by treating Li with SnF_2_ and bare Li. Voltage profiles show that the best Li deposition behavior and longest plating/stripping cycles were achieved by AFH-25. The AFH-25 fully protects the Li electrode as well as renders a uniform, smooth, and dendrite-free Li deposition compared to AFH-10, and AFH-55 (Supplementary Fig. 2a–c). Further, to find the best SEI layer for the Li metal electrode protection, various characterizations have been conducted on the SEI layers from different SnF_2_ concentrations. The impedance measurements were carried out to calculate the charge transfer resistance (*R*_ct_) of symmetrical cells. Figure 3b shows the Nyquist plot of bare Li and, AFH-10, AFH-25, and AFH-55, respectively. The impedance results were fitted using an equivalent circuit as shown in Supplementary Fig. 2d, e.

The bare Li anode has no artificial SEI before Li plating/stripping cycling. Although there is in situ SEI formation when the bare Li contacts the electrolyte before Li plating/stripping cycling, such SEI is very thin and does not fully cover the surface of the Li electrode. This SEI acts as an insignificant interfacial resistance layer^19^; therefore, the bare Li symmetrical cell before Li plating/stripping cycling just has one Electrochemical Impedance Spectroscopy (EIS) semicircle (Fig. 3b). The single semicircle indicates the *R*_ct_ between the bare Li electrode and electrolyte^33^. However, after Li plating/stripping cycles, a much thicker SEI (Supplementary Fig. 10a) is formed and fully covers the bare Li. This supports the presence of two semicircles (Fig. 3c, d) in bare Li after 10 cycles and 50 cycles. The slightly higher *R*_s_ of SnF_2_–Li symmetrical cell compared to bare Li symmetrical cell at fresh conditions can be attributed to an artificial SEI film of SnF_2_-treated Li^34^. The higher *R*_ct_ in bare Li symmetrical cell before plating stripping cycles can be attributed to the lower electrolyte wettability of the Li electrode that leads to sluggish Li-ion transport^19,35^. In addition, the absence of a protective layer to inhibit the possible side reactions leads to electrolyte consumption, and the formation of unstable and fragile SEI^19,25,35^. Thus, the cell’s impedance increases.

In contrast, AFH-10, AFH-25, and AFH-55 symmetrical cells show two semicircles. The first semicircle in the higher frequency range indicates the interfacial resistance of the artificial SEI or resistance of Li-ion flux through an artificial SEI, and the second semicircle in the lower frequency range indicates the *R*_ct_ between the artificial SEI and the electrolyte^19,33,36–39^. The lower *R*_ct_ of AFH-10, AFH-25, and AFH-55 symmetrical cells can be attributed to the better electrolyte wettability of the artificial SEI, the effective control of side reactions, and the stabilized SEI^35,40,41^. In addition, the LiF in the SEI layer has high Li-ion conductivity, low diffusion barrier, and high surface energy. These allow sufficient Li-ion transport that lowers the *R*_ct_^17,18,40,41^. The symmetrical cells based on the AFH-25 anode exhibit the least *R*_ct_ value of 47 Ω, which can be attributed to the fast Li-ion transport with an optimized SEI thickness of 25 µm. All the quantified impedance results are summarized in Supplementary Table 1. Thus, 25 µm is considered as the optimal thickness of SEI. Further measurements, characterizations, and comparisons were based on the bare Li and AFH-25 unless stated. The stability of usual SEI in bare Li and AFH-25 in protected Li was measured using a Nyquist plot as a function of time (Supplementary Fig. 2f, g) and impedance results are listed in Supplementary Table 2. In bare Li, the *R*_ct_ of the symmetrical cell increases continuously from 343.50 Ω at fresh conditions to 1717.00 Ω at 600 h (hours). In contrast, the AFH-25 symmetrical cell shows a gradual increment in *R*_ct_ and remains steady, i.e., 47.00 Ω at the fresh condition and remains steady around 75.75 Ω at 600 h.

To investigate the stability of the SEI, impedance measurements of bare Li and AFH-25 were carried out after 10 cycles and 50 cycles of plating/stripping as shown in Fig. 3c, d. The *R*_s_ of bare Li increases from 2.39 Ω at the fresh condition to ~28 Ω after 10 cycles, and ~ 33 Ω after 50 cycles. The *R*_s_ of AFH-25 increases from 9.59 Ω at fresh to ~18 Ω after 10 cycles, and ~22 Ω after 50 cycles. The higher value of *R*_s_ in the bare Li compared to AFH-25 indicates a higher amount of electrolyte consumption in bare Li due to the formation of Li dendrites with a high surface area, formation/deformation of SEI^42^, and other inactive products formed from side reactions^43^. After 10 cycles of plating/stripping, the *R*_ct_ of bare Li and AFH-25 were ~88.43 Ω and ~48.04 Ω, respectively. After 50 cycles of plating/stripping cycles, *R*_ct_ of bare Li and AFH-25 reduced to ~74.61 Ω and ~37.62 Ω, respectively. The decrease in *R*_ct_ of bare Li after 10 cycles and 50 cycles can be attributed to the higher surface area of Li dendrite that allows more electrolyte contact, and dissolution of the passivation film^44^. However, the excessive consumption of electrolytes ultimately leads to electrolyte dry out causing premature cell failure^32^. In contrast, the decrease in *R*_ct_ after the 10 cycles and 50 cycles in AFH-25 can be attributed to the stabilization of artificial SEI. The AFH-25 provides higher electrolyte wettability, higher Li-ion conductivity, lower diffusion barrier, and higher surface energy. These allow sufficient Li-ion transport that lowers the *R*_ct_^17,18^. In addition, the AFH-25 prevents the direct contact of electrolyte and Li electrode to inhibit the reaction between Li and electrolyte. Moreover, the Sn–Li alloy is responsible to lower the Li-ion diffusion barrier for improving the Li metal interphase stability^25–27,45–47^. Further, the Li-ion transference number (T_Li_^+^) in the absence of an artificial layer was calculated to be 0.43 and with AFH-25, the value of T_Li_^+^ increased to 0.52 (calculated from Supplementary Fig. 3). The increase in T_Li_^+^ can be attributed to the increase in Li^+^ fraction by the dissociation of ion pairs^48^. This demonstrates that the artificial SEI facilitates fast movement of Li-ion and is beneficial for enhanced cycling performance^22,24^.

### Materials characterizations

X-ray photoelectron spectrum (XPS) measurement was employed to investigate the chemical composition on the SEI surface of AFH-25 anode. Supplementary Fig. 4a shows two main peaks at 487.70 eV and 496.01 eV that can be assigned to Sn 2d_5/2_ and Sn 3d_5/2_, respectively, indicating the presence of Sn in an artificial SEI^25,49^. Sn as an SEI constituent stores Li by alloying reaction to form Li_5_Sn_2_^25,50^. The single Li 1 s peak at 55.79 eV (Supplementary Fig. 4b) and the single F 1 s peak at 684.96 eV (Supplementary Fig. 4c) correspond to the presence of LiF^18,20^. The LiF as a component of SEI regulates uniform Li plating/stripping^20,21,51^.

Atomic force microscopy (AFM) was performed to observe surface topography and measure the corresponding Young’s modulus of bare Li and AFH-25 as shown in Supplementary Fig. 5a–d. The average root mean square (r.m.s.) roughness value of bare Li and AFH-55 was 260 nm and 38 nm, respectively. Higher roughness indicates the uneven surfaces that can create large protuberance responsible for uneven Li deposition^52^. In contrast, the smooth surface of protected Li renders homogenous Li deposition. The corresponding Young’s modulus mapping of bare Li and AFH-25 shows an average Young’s modulus value of 0.28 GPa and 55.60 GPa, respectively. This high value of Young’s modulus can be attributed to the contribution of all SEI components (LiF, Sn–Li, and Sn). Due to the strong ionic bond between Li and F, LiF shows Young’s modulus value ranging from 50 to 140 GPa^53–55^. The B1 crystal structure of LiF (similar to NaCl type) remains invariant under high pressure up to ~100 GPa and high temperature up to the melting point^56^. In addition, Sn is considered as a mechanically robust and highly stable material^57,58^. Based on the crystal orientation, the theoretical range for Young’s modulus of Sn varies from 26.30 to 84.70 GPa^57,59–61^ and that of Li_5_Sn_2_ is from 40.96 to 74.20 GPa^57,62^.

To better understand the mechanism of the superior performance of fluorinated artificial SEI layers, contact angle and transference number measurements were conducted. The contact angle measurement was found to be 30° for bare Li and 1° for AFH-25 as shown in Supplementary Fig. 6a, b. This higher electrolyte affinity leads to a higher surface energy of the SEI layer that facilitates fast Li-ion diffusion and nucleation. In addition, linear sweep voltammetry shows that the bare Li has a steeper slope than the AFH-25 (Supplementary Fig. 6c). This implies that the artificial SEI protective layer of Li lowers the electronic conductivity. The electronic resistive nature of SEI is favorable to first deposit/plate Li underneath the SEI. Thus, even with a high current density and large deposition amount of Li, we can still lower the local current density in order to homogenize the Li-ion distribution in the SEI layer. The growth of Li dendrite is also inhibited due to the semiconducting nature of SEI^32^. In this work, the ionic conductivity of the protected Li was calculated to be 5.84 × 10^−4^ S cm^−1^ (Supplementary Fig. 6d). This value of ionic conductivity is large enough to diffuse Li-ion^37^.

### Electrochemical performance, elemental composition, and Li deposition morphology

**Fig. 4:**
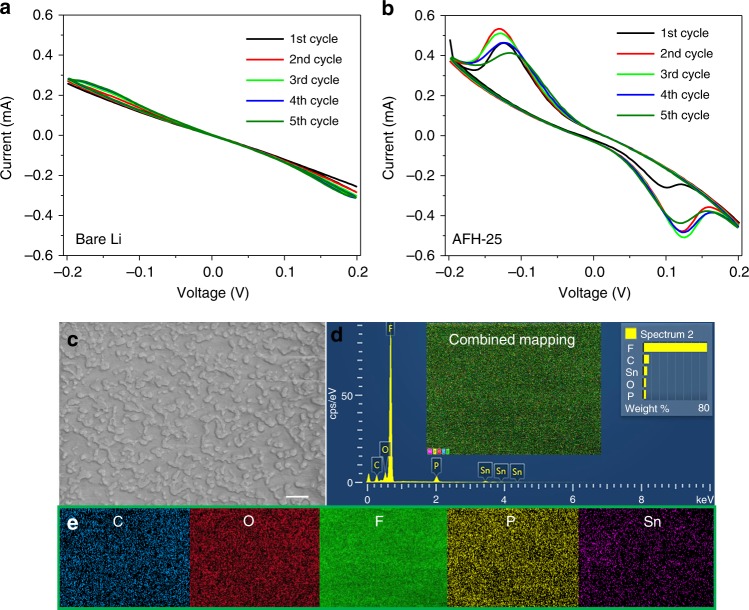
Electrochemical performance and high-resolution SEM imaging. **a**, **b** CV measurements of bare Li and AFH-25 symmetrical cells, respectively. **c** The high-resolution SEM image of AFH-25. The scale bar is 2 μm. **d** The corresponding EDS of the aforementioned area. **e** The corresponding elemental mapping of the aforementioned area.

To understand the electrochemical properties of the SEI, cyclic voltammetry (CV) measurements of bare Li, and AFH-25 symmetrical cells were investigated (Fig. 4a, b). The bare Li symmetrical cell showed almost a straight line indicating the Li plating/stripping. In contrast, the AFH-25 symmetrical cell showed broad peaks at ~0.12 V and at ~−0.12 V over multiple cycles in addition to the typical Li/Li^+^ polarization curves, confirming the occurrence of lithiation/delithiation of Tin (Sn) and Li plating/stripping underneath the SEI. This indicates that the electrochemically active Sn can reversibly store Li by the formation of Sn–Li alloy^25^. A similar observation of sodiation/desodiation of Sn was reported in Na-metal battery, suggesting the favorable reaction mechanism of the Sn-based SEI^45^. To observe the distribution of elements present on the surface of AFH-25, the energy dispersive spectrum (EDS) elemental mapping was carried out. The zoom-in SEM image and its corresponding EDS elemental mapping are shown in Fig. 4c–e. The zoom-in SEM image (Fig. 4c) shows that the artificial fluorinated hybrid SEI is composed of compactly stacked microstructures^20^. The inset image of EDS (Fig. 4d) is the combined elemental mapping. The elemental mapping of surface morphology (Fig. 4e) shows that the elements C, O, F, and Sn are uniformly distributed.

The EDS elemental mapping (Fig. 4e) shows that F and Sn are uniformly distributed on the SEI. Thus, the compact surface morphology (Fig. 4c) of LiF, Sn–Li alloy and Sn contribute to the total surface strength. This Young’s modulus value is significantly high to suppress the Li dendrite growth^63^, which indicates the artificial layer composed of Sn, LiF, and Sn–Li alloy can withstand physical changes, provide sufficient mechanical stability during Li plating /stripping, and offer high resistance/strength to suppress Li dendrite growth^20,31,37,63–69^.

**Fig. 5:**
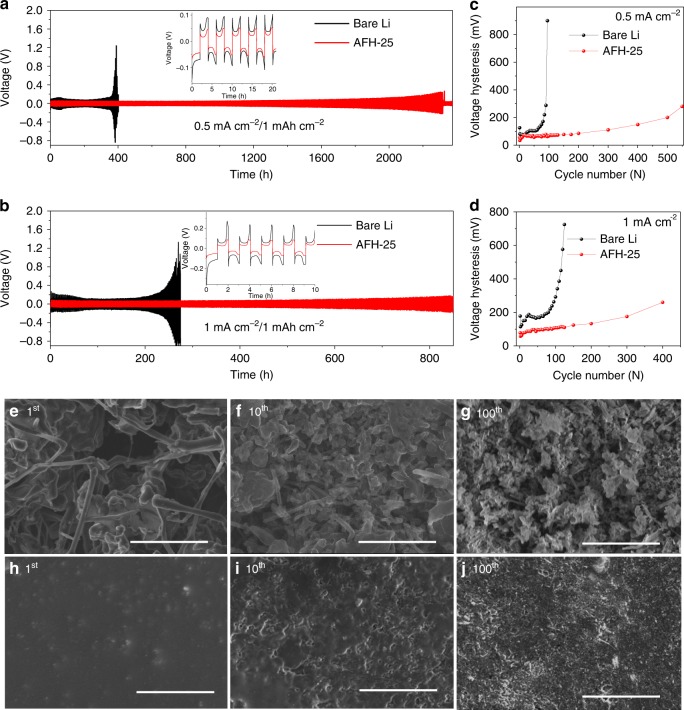
Electrochemical symmetrical cell test and morphological analysis. **a**, **b** Plating/stripping voltage profile of symmetrical cells at 0.5 mA cm^−2^ and 1 mA cm^−2^, respectively. The insets are the first five plating/stripping cycles. **c**, **d** The corresponding average voltage hysteresis from **a** and **b**, respectively. **e**–**j** The SEM images of bare Li **e**–**g** and AFH-25 **h**–**j** at 1st, 10th, and 100^th^ plating, respectively. The scale bars are 20 μm.

The plating/stripping voltage profile of bare Li and AFH-25 was carried out to investigate interfacial stability. Figure 5a, b shows the voltage profile of Li plating/stripping of bare Li and AFH-25 symmetrical cells that achieved a capacity of 1 mAh cm^−2^ at a current density of 0.5 mA cm^−2^ and 1 mA cm^−2^, respectively. The AFH-25 symmetrical cells show a longer plating/stripping cycles than the bare Li. In bare Li, the overpotential increases continuously that leads to the early death of cell at ~400 h and ~250 h at 0.5 mA cm^−2^ and 1 mA cm^−2^, respectively. In contrast, AFH-25 symmetrical cells show a stable voltage profile for longer plating/stripping cycles. The AFH-25 symmetrical cell can run up to ~2325 h and ~850 h of plating/stripping at 0.5 mA cm^−2^ and 1 mA cm^−2^, respectively. In bare Li, the reaction between the electrolyte and Li electrode, growth of Li dendrite due to nonuniform Li deposition after plating/stripping, and formation of fragile/unstable SEI can lead to electrolyte dry out^32^. Eventually, the overpotential occurs and causes the premature death of the cell. In contrast, the artificial SEI, formed by treating with SnF_2_, physically protects the Li from side reactions with the electrolyte and provides a route for uniform Li deposition^65^. In addition, the SEI component such as Sn can reversibly store Li by alloying. The presence of LiF in the protective SEI layer facilitates uniform Li diffusion to reversibly store Li by plating on the Li electrode underneath the SEI^18,19,27^. The nucleation overpotential is the magnitude of the voltage spike at the onset of Li deposition as shown in the first five cycles of plating/stripping (insets of Fig. 5a, b). The nucleation overpotential of AFH-25 is lower than the bare Li. The Sn and Li–Sn alloy in the artificial SEI provide uniform dispersive seeds as a nucleation site for Li deposition. This contributes to the uniform distribution of Li ions for a homogeneous Li deposition and longer plating/stripping cycles. The plating/stripping hours of SnF_2_-treated artificial SEI have been improved and compared to the previously reported literature as summarized in Supplementary Table 3. It is noticed that AFH-25 symmetrical cell shows remarkable long plating/stripping performance with reduced voltage overpotential. Figure 5c, d shows the average voltage hysteresis that is the difference between the voltage of Li stripping and plating. This is mainly dependent on the current density and nature of interfacial SEI. The voltage hysteresis of AFH-25 exhibits a more stable and less fluctuating voltage curve compared to the bare Li. The artificial SEI composed of Sn, LiF, and Sn–Li alloy lowers the practical current density and reduces the *R*_ct_. The reduced hysteresis in AFH-25 symmetric cells indicates a low-voltage polarization voltage profile in the SnF_2_-protected Li/NMC111 full cell. Similar improved voltage–time profiles were obtained when plated/stripped at other higher constant current density rates (Supplementary Fig. 7a–c) and different current density rates (Supplementary Fig. 8a, b).

SEM was performed to study the morphology of Li deposition on bare Li and AFH-25. Figure 5e–j shows the SEM images of bare Li and AFH-25 after 1st, 10th, and 100th plating at 0.5 mA cm^−2^ with a capacity of 1 mAh cm^−2^. The bare Li (Fig. 5e–g) exhibits a large roughness surface with Li dendrites and filament protruding out in random orientations, suggesting nonuniform Li deposition and uncontrolled growth of Li dendrites. This leads to dead and mossy Li upon cycling on bare Li, attributed to the continuous corrosion of reactive Li and electrolyte consumption. These cause the whim of increased voltage and impedance. The sharp spikes of Li dendrites can pierce the separator threatening safety. As a result, premature failure of the cell is more pronounced. In contrast, AFH-25 (Fig. 5h–j) exhibits a flat, smooth surface without obvious dendritic or mossy Li. The compact and uniform artificial layer serves as a physical protection barrier to inhibit the penetration of organic electrolyte and subsequent corrosion of the underlying Li electrode. In addition, the SEI components Sn and Sn–Li alloy reversibly store Li by alloying as Li_5_Sn_2_. The insulating SEI component LiF is unfavorable for Li nucleation that facilitates Li-ion diffusion and store Li by plating on the Li electrode underneath, which leads to a uniform and smooth Li deposition^17–22^. Moreover, Young’s modulus (55.60 GPa) of the artificial SEI layer is much higher than the threshold value of 6 GPa to suppress the growing Li dendrites^63^. Therefore, an extremely long and stable cycling performance with reduced overpotential has been achieved.

To study the composition and distribution of elements on the surface of bare Li and AFH-25 after 1st plating at 0.5 mA cm^−2^, EDS and elemental mapping were performed (Supplementary Fig. 9a, b). The top-view SEM image of AFH-25 has 28.92 wt% fluorine (F), which is higher than the bare Li at 20.92 wt%. This verifies that excess fluorine came from the dissociation of SnF_2_ anions. In addition, the top-view SEM image showed that AFH-25 has uniform elemental mapping distribution of C, O, F, P, and Sn, suggesting a uniform coverage of SEI components. In contrast, the bare Li shows a nonuniform elemental mapping distribution and inhomogeneous Li deposition. Supplementary Fig. 10a, b shows the cross-sectional SEM images of the bare Li and AFH-25 after 100th plating at 0.5 mA cm^−2^. The SEI thickness increases from 0 µm to ~120 µm in bare Li and from 25 µm to ~60 µm for AFH-25 after 100th plating. On the bare Li, the irreversibly plated Li in each cycle forms an unstable, thicker, and insulating SEI layer retarding the Li-ion transport. The gradually increasing thickness of the SEI layer further increases the cell’s impedances and eventually causes the cell to die^20^. However, in the AFH-25, a thinner and denser layer of SEI was observed due to the stable SEI as well as effective control of consumption of Li and electrolyte^32^.

### Battery performance

**Fig. 6:**
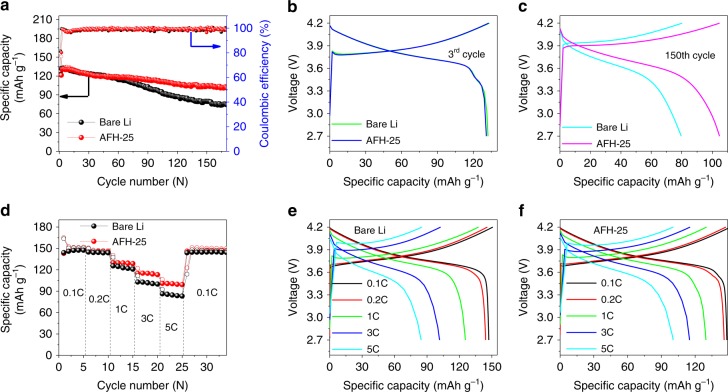
Electrochemical performance comparison of the cells using bare Li and AFH-25 anode. **a** Long-term cycling performance of batteries at a current density of 1 C. **b**, **c** Charge/discharge voltage profiles of batteries at a current density of 1 C. **d** Rate capability comparison of NMC111 coupled with bare Li and AFH-25 anode. **e**, **f** Charge/discharge voltage profiles of NMC111 coupled with bare Li and AFH-25 anode, respectively at different current density rates.

To evaluate the potential application and feasibility of AFH-25 anode in practical batteries, NMC111 cathode (loading = 11.88 mg cm^−2^) was adopted to assemble a full LMB. Figure 6a shows the cycling performance of full cell using bare Li and AFH-25 as an anode at a constant current density of 1 C. The initial 3rd discharge capacity of AFH-25/NMC111 cell is 130.86 mAh g^−1^ and still remains 104.71 mAh g^−1^ at 150th cycles, retaining 80.01%. In contrast, the 3rd specific discharge capacity of bare Li/NMC111 full cell shows a sharp decrease from 132.20  mAh g^−1^ to 79.59 mAh g^−1^, retaining 60.20% at the 150th cycle. The superior discharge capacity in long-term cycling demonstrates the SnF_2_ treatment is an effective way to achieve outstanding cell stability. The bare Li/NMC111 and AFH-25/NMC111 show the 1st CE of 81.33% and 83.31%, respectively. The lower CE in bare Li/NMC111 can be attributed to the side reactions and formation of unstable and fragile SEI, while AFH-25/NMC111 exhibits stabilized SEI. With higher cycling, the CE in both full cells remains above ~99%. In addition, the cycling performance of artificially fluorinated hybrid protected Li anode at a lower N/P ratio of 2:1 was studied (details in Supplementary Fig. 11). The NMC111 cathode coupled with protected Li anode shows improved cycling performance compared to the unprotected Li. Electrochemical voltage profiles (Fig. 6b, c) show that polarizing voltages of the full cell using the bare Li and AFH-25 anode is almost the same at the beginning cycles. The rapid increase in polarizing voltages with cycling (Fig. 6b) as observed in the bare Li was attributed to the unstable SEI. However, a significantly reduced overpotential was observed (Fig. 6c) with AFH-25/NMC111 configuration even after long cycles (e.g., the 150th cycle) due to the stabilized SEI formation.

Figure 6d shows the cycling performance of full cells at different current rates. At a higher rate of 5 C, the specific charge/discharge capacity at the 23rd cycle using AFH-25 and bare Li was 100.54/100.44 mAh g^−1^ and 85.29/84.60 mAh g^−1^, respectively. The capacities at different rates are summarized in Supplementary Table 4. The higher capacity at higher rates was achieved using AFH-25 than bare Li as an anode. This is in good agreement with symmetrical cell tests and impedance measurements. Figure 6e, f shows the corresponding charge/discharge voltage profiles at different rates. The significant reduction in voltage hysteresis was observed using AFH-25 as an anode. This is attributed to the stabilized artificial SEI of AFH-25 that effectively prevents the parasitic reactions between Li and electrolyte and render uniform dendrite-free Li deposition^17^. On one hand, LiF is poor in electronic conductivity to prevent electron flow, which suppresses Li dendrite growth^16^. On the other hand, LiF has high ionic conductivity, low diffusing energy, and high surface energy, which allow sufficient Li-ion diffusion during plating^17,18^. Thus, a uniform and dendrite-free morphology of electrodeposited Li was expected on AFH-25 anode. In addition, the Li storage mechanism by forming reversible Sn–Li alloy and Li plating provides a promising route to develop a stabilized SEI layer^25^. However, the sharp capacity decay in bare Li/NMC111 is attributed to the failure of the conductive framework in the anode induced by the highly resistive, fragile, and unstable SEI formation, and dead Li covering Li anode^24^. The formation of unstable SEI, loss of Li, and electrolyte consumption due to the high surface area of Li dendrites cause the capacity fading and low CE in the bare Li devices^36^. Supplementary Fig. 12a, b shows the Nyquist plots of the full cells using NMC111 as a cathode, bare Li and AFH-25 as an anode, respectively. The *R*_ct_ of the cell using bare Li and AFH-25 as the anode is 189.00 Ω and 12.91 Ω, respectively. The largely reduced *R*_ct_ of AFH-25 indicates improvement in charge transfer kinetics^50^. This is attributed to the stabilized SEI consisting of LiF, Sn, and Sn–Li alloy, which allows sufficient Li-ion diffusion and inhibits undesired side reactions^21,25^. Supplementary Fig. 12c shows the equivalent circuit for fitting. Impedance results are summarized in Supplementary Table 5.

## Discussion

An artificial SEI consisting of LiF, Sn, and Sn–Li alloy was constructed by treating Li anode with SnF_2_-containing electrolyte, whose chemical and mechanical stability protects the Li from the side reactions and suppresses the Li dendrite growth while allowing for fast Li^+^ transport and reversible Li–Sn alloying. Outstanding electrochemical performances were achieved in both Li//Li symmetric and full Li-metal cells based on an NMC111 cathode, as evidenced by long-term plating/stripping stability (~2325 h), reduced overpotential in the former and excellent cycling stability, high capacity retention of 80.01% and high specific charge/discharge capacity in the latter. This effective approach to Li-stabilization based on SnF_2_-induced interphase opens an alternative door to the development of high energy density storage devices using not only transition metal oxide cathodes but also emerging cathode chemistries, such as Li–S and Li–O_2_.

## Methods

### Materials and preparations

Li chips with a diameter of 15.6 mm and thickness 250 µm were purchased from Xiamen Tmax, China. Tin (II) fluoride was purchased from Acros Organics. The surface of Li chips was cleaned and polished with a sharp blade in order to remove the impurities and oxide layer. Different wt% of SnF_2_ was mixed in 1 M LiPF_6_ in a mixture solvent of ethylene carbonate (EC)/diethylcarbonate (DEC; 1:1 v/v) and partially dissolved SnF_2_ solution was ultrasonicated each time before drop-casting. A volume of 30 µL of electrolyte-containing different wt% SnF_2_ was dropped cast on the surface of Li that changed from the silver shiny color to immediate dark gray. After drying for ~48 h at 60 °C inside an Ar glove box, the surface becomes whitish color and the surface of the electrodes were rinsed with dimethyl carbonate solvent to remove any residues. Both the bare Li and the Li treated with SnF_2_ were cut into a 12 mm circular disc.

### Electrode fabrication

The cathode used was Li nickel cobalt manganese oxide (LiNi_1/3_Co_1/3_Mn_1/3_O_2_) or (NMC111), with the areal mass loading of the electrode 11.88 mg cm^−2^ and active material 9.98 mg cm^−2^. The diameter of the cathode electrodes was 12 mm.

### Materials characterizations

A sealed container was used to avoid direct contact with moisture or air while transferring the samples from the glove box during material characterizations. SEM images, EDS, and elemental mapping were performed using Hitachi S-3400N SEM and Hitachi S-4700N FESEM. The XRD was conducted using a Rigaku SmartLab diffractometer. All the samples were encapsulated with Kapton tape during XRD measurement to avoid moisture contamination. XPS was performed on the Thermo Scientific X-ray Photoelectron Spectrometer with Al Ka radiation. The surface morphology and Young’s modulus measurement of bare Li and SnF_2_-treated Li was carried out using Bruker AFM equipped with the MAC III controller using RTESPA-525 tip with a resonant frequency of 75 kHz through quantitative nanomechanical mode. The contact angle was measured by VCA2000 video contact angle system.

### Electrochemical characterization

Symmetric cells and full cells were assembled in the Ar glove box using bare Li and protected Li as anode using CR-2032 coin-type cells. A volume of 60 µL of 1 M LiPF_6_ in the mixture of EC/DEC (1:1 v/v) was used as the electrolyte and Celgard 2500 film of 25 µm thickness as the separator. The galvanostatic charge–discharge measurements of the coin cells were carried out using the LAND CT2001A system. Plating/stripping of the symmetrical cells was performed at various areal current density from 0.5 to 5 mA cm^−2^ to achieve various areal capacities from 1 to 3 mAh cm^−2^. Full cells were cycled between 2.7 V to 4.2 V at a constant current density of 1 C and at various current density rates from 0.1 C, 0.2 C, 1 C, 3 C, and 5 C for every five cycles and followed back to 0.1 C. EIS measurement was conducted by an electrochemical workstation (Ametek VERSATAT3-200 potentiostat) with a 10 mV amplitude AC signal with frequency ranging from 100 kHz to 0.1 Hz.

## Supplementary information

Supplementary Information

## Data Availability

The authors declare that all the data supporting the findings of this study are available within the article and its Supplementary Information or from the corresponding author upon reasonable request.

## References

[1] Bruce PG, Freunberger SA, Hardwick LJ, Tarascon JM (2012). Li-O_2_ and Li-S batteries with high energy storage. Nat. Mater..

[2] Gurung A, Qiao Q (2018). Solar charging batteries: advances, challenges, and opportunities. Joule.

[3] Gurung A (2017). Highly efficient perovskite solar cell photocharging of lithium ion battery using DC–DC booster. Adv. Energy Mater..

[4] Zhou Z (2017). Binder free hierarchical mesoporous carbon foam for high performance lithium ion battery. Sci. Rep..

[5] Harry KJ, Hallinan DT, Parkinson DY, MacDowell AA, Balsara NP (2014). Detection of subsurface structures underneath dendrites formed on cycled lithium metal electrodes. Nat. Mater..

[6] Albertus P, Babinec S, Litzelman S, Newman A (2018). Status and challenges in enabling the lithium metal electrode for high-energy and low-cost rechargeable batteries. Nat. Energy.

[7] Yang CP, Yin YX, Zhang SF, Li NW, Guo YG (2015). Accommodating lithium into 3D current collectors with a submicron skeleton towards long-life lithium metal anodes. Nat. Commun..

[8] Wu H, Zhuo D, Kong D, Cui Y (2014). Improving battery safety by early detection of internal shorting with a bifunctional separator. Nat. Commun..

[9] Liu K (2017). Extending the life of lithium-based rechargeable batteries by reaction of lithium dendrites with a novel silica nanoparticle sandwiched separator. Adv. Mater..

[10] Jin Y (2018). An intermediate temperature garnet-type solid electrolyte-based molten lithium battery for grid energy storage. Nat. Energy.

[11] Naderi R (2017). Activation of passive nanofillers in composite polymer electrolyte for higher performance lithium-ion batteries. Adv. Sustain. Syst..

[12] McGraw M (2016). One-step solid-state in-situ thermal polymerization of silicon-PEDOT nanocomposites for the application in lithium-ion battery anodes. Polym. (Guildf.)..

[13] Liu Y (2018). An ultrastrong double-layer nanodiamond interface for stable lithium metal anodes. Joule.

[14] Lin D (2017). Three-dimensional stable lithium metal anode with nanoscale lithium islands embedded in ionically conductive solid matrix. Proc. Natl Acad. Sci. USA.

[15] Ding F (2013). Dendrite-free lithium deposition via self-healing electrostatic shield mechanism. J. Am. Chem. Soc..

[16] Pan J, Cheng YT, Qi Y (2015). General method to predict voltage-dependent ionic conduction in a solid electrolyte coating on electrodes. Phys. Rev. B.

[17] Lu Y, Tu Z, Archer LA (2014). Stable lithium electrodeposition in liquid and nanoporous solid electrolytes. Nat. Mater..

[18] Yuan Y (2019). Regulating Li deposition by constructing LiF-rich host for dendrite-free lithium metal anode. Energy Storage Mater..

[19] Choudhury S, Archer LA (2016). Lithium fluoride additives for stable cycling of lithium batteries at high current densities. Adv. Electron. Mater..

[20] Yan C (2018). An armored mixed conductor interphase on a dendrite-free lithium-metal anode. Adv. Mater..

[21] Zhao J (2017). Surface fluorination of reactive battery anode materials for enhanced stability. J. Am. Chem. Soc..

[22] Fan X (2018). Fluorinated solid electrolyte interphase enables highly reversible solid-state Li metal battery. Sci. Adv..

[23] Kanamura K, Shiraishi S, Takehara ZI (1996). Electrochemical deposition of very smooth lithium using nonaqueous electrolytes containing HF. J. Electrochem. Soc..

[24] Zhang XQ, Cheng XB, Chen X, Yan C, Zhang Q (2017). Fluoroethylene carbonate additives to render uniform Li deposits in lithium metal batteries. Adv. Funct. Mater..

[25] Tu Z (2018). Fast ion transport at solid–solid interfaces in hybrid battery anodes. Nat. Energy.

[26] Choudhury S (2017). Electroless formation of hybrid lithium anodes for fast interfacial ion transport. Angew. Chem. Int. Ed..

[27] Liang X (2017). A facile surface chemistry route to a stabilized lithium metal anode. Nat. Energy.

[28] Yan K (2016). Selective deposition and stable encapsulation of lithium through heterogeneous seeded growth. Nat. Energy.

[29] Zhang L, Zhang K, Shi Z, Zhang S (2017). LiF as an artificial SEI layer to enhance the high-temperature cycle performance of Li_4_Ti_5_O_12_. Langmuir.

[30] Seyring M (2018). Phase formation in the ternary systems Li-Sn-C and Li-Sn-Si. Thermochim. Acta.

[31] Lin D (2016). Layered reduced graphene oxide with nanoscale interlayer gaps as a stable host for lithium metal anodes. Nat. Nanotechnol..

[32] Pathak R (2019). Ultrathin bilayer of graphite/SiO_2_ as solid interface for reviving Li metal anode. Adv. Energy Mater..

[33] Gaikwad AM (2015). A high areal capacity flexible lithium-ion battery with a strain-compliant design. Adv. Energy Mater..

[34] Chen Z (2016). Fast and reversible thermoresponsive polymer switching materials for safer batteries. Nat. Energy.

[35] Wu F (2018). Comparison of performance and optoelectronic processes in ZnO and TiO_2_ nanorod array-based hybrid solar cells. Appl. Surf. Sci..

[36] Cha E (2018). 2D MoS_2_ as an efficient protective layer for lithium metal anodes in high-performance Li-S batteries. Nat. Nanotechnol..

[37] Liu Y (2017). An artificial solid electrolyte interphase with high Li-ion conductivity, mechanical strength, and flexibility for stable lithium metal anodes. Adv. Mater..

[38] Li NW, Yin YX, Yang CP, Guo YG (2016). An artificial solid electrolyte interphase layer for stable lithium metal anodes. Adv. Mater..

[39] Chen K (2019). Flower-shaped lithium nitride as a protective layer via facile plasma activation for stable lithium metal anodes. Energy Storage Mater..

[40] Gunceler D, Letchworth-Weaver K, Sundararaman R, Schwarz KA, Arias T (2013). The importance of nonlinear fluid response in joint density-functional theory studies of battery systems. Modell. Simul. Mater. Sci. Eng..

[41] Ma L, Kim MS, Archer LA (2017). Stable artificial solid electrolyte interphases for lithium batteries. Chem. Mater..

[42] Chen Q (2019). Electrochemically induced highly ion conductive porous scaffolds to stabilize lithium deposition for lithium metal anodes. J. Mater. Chem. A.

[43] Ouyang M (2015). Low temperature aging mechanism identification and lithium deposition in a large format lithium iron phosphate battery for different charge profiles. J. Power Source.

[44] An Y (2018). Vacuum distillation derived 3D porous current collector for stable lithium-metal batteries. Nano Energy.

[45] Zheng X (2019). Toward a stable sodium metal anode in carbonate electrolyte: a compact, inorganic alloy interface. J. Phys. Chem. Lett..

[46] Zhang SS, Fan X, Wang C (2017). A tin-plated copper substrate for efficient cycling of lithium metal in an anode-free rechargeable lithium battery. Electrochim. Acta.

[47] Xu Q, Yang Y, Shao H (2015). Substrate effects on Li^+^ electrodeposition in Li secondary batteries with a competitive kinetics model. Phys. Chem. Chem. Phys..

[48] Liu W (2017). Enhancing ionic conductivity in composite polymer electrolytes with well-aligned ceramic nanowires. Nat. Energy.

[49] Gu C, Mai Y, Zhou J, You Y, Tu J (2012). Non-aqueous electrodeposition of porous tin-based film as an anode for lithium-ion battery. J. Power Source.

[50] Pathak R (2018). Self-recovery in Li-metal hybrid lithium-ion batteries via WO_3_ reduction. Nanoscale.

[51] Wang H, Lin D, Liu Y, Li Y, Cui Y (2017). Ultrahigh-current density anodes with interconnected Li metal reservoir through overlithiation of mesoporous AlF_3_ framework. Sci. Adv..

[52] Li C (2019). Two-dimensional molecular brush-functionalized porous bilayer composite separators toward ultrastable high-current density lithium metal anodes. Nat. Commun..

[53] Shin H, Park J, Han S, Sastry AM, Lu W (2015). Component-/structure-dependent elasticity of solid electrolyte interphase layer in Li-ion batteries: experimental and computational studies. J. Power Source.

[54] Dong H (2014). Compression of lithium fluoride to 92 GPa. High. Press. Res..

[55] Zhang X (2019). Self-suppression of lithium dendrite in all-solid-state lithium metal batteries with poly (vinylidene difluoride)-based solid electrolytes. Adv. Mater..

[56] Smirnov N (2011). Ab initio calculations of the thermodynamic properties of LiF crystal. Phys. Rev. B.

[57] Stournara ME, Guduru PR, Shenoy VB (2012). Elastic behavior of crystalline Li-Sn phases with increasing Li concentration. J. Power Source.

[58] Qaiser N, Kim YJ, Hong CS, Han SM (2016). Numerical modeling of fracture-resistant Sn micropillars as anode for lithium ion batteries. J. Phys. Chem. C.

[59] Chen J (2007). Mechanical analysis and in situ structural and morphological evaluation of Ni–Sn alloy anodes for Li ion batteries. J. Phys. D Appl. Phys..

[60] Wang J, Chen-Wiegart YCK, Wang J (2014). In situ three-dimensional synchrotron X-ray nanotomography of the (De) lithiation processes in tin anodes. Angew. Chem. Int. Ed..

[61] Mukaibo H, Momma T, Shacham-Diamand Y, Osaka T, Kodaira M (2007). In situ stress transition observations of electrodeposited Sn-based anode materials for lithium-ion secondary batteries. Electrochem. Solid-State Lett..

[62] Chou C-Y, Lee M, Hwang GS (2015). A comparative first-principles study on sodiation of silicon, germanium, and tin for sodium-ion batteries. J. Phys. Chem. C.

[63] Xu R (2018). Artificial soft-rigid protective layer for dendrite-free lithium metal anode. Adv. Funct. Mater..

[64] Zheng G (2014). Interconnected hollow carbon nanospheres for stable lithium metal anodes. Nat. Nanotechnol..

[65] Wang M (2018). Effect of LiFSI concentrations to form thickness-and modulus-controlled SEI layers on lithium metal anodes. J. Phys. Chem. C.

[66] Lee H, Lee DJ, Kim YJ, Park JK, Kim HT (2015). A simple composite protective layer coating that enhances the cycling stability of lithium metal batteries. J. Power Source.

[67] Zhu B (2017). Poly (dimethylsiloxane) thin film as a stable interfacial layer for high-performance lithium-metal battery anodes. Adv. Mater..

[68] Li B, Wang Y, Yang S (2018). A material perspective of rechargeable metalliclithium anodes. Adv. Energy Mater..

[69] Chai J (2018). Dendrite-free lithium deposition via flexible-rigid coupling composite network for LiNi_0.5_Mn_1. 5_O_4_/Li metal batteries. Small.

