# Supplementary material for: Fluorinated hybrid solid-electrolyte-interphase for dendrite-free lithium deposition
Source: Nat Commun. 2020 Jan 3;11:93. doi: 10.1038/s41467-019-13774-2 (PMC6941966; doi:10.1038/s41467-019-13774-2)
Supplement: Supplementary file 1 — Supplementary Information [file 41467_2019_13774_MOESM1_ESM.pdf]

Supplementary Information

Fluorinated Hybrid Solid-Electrolyte-Interphase for Dendrite-Free Lithium Deposition

Pathak et al.

Supplementary Figures

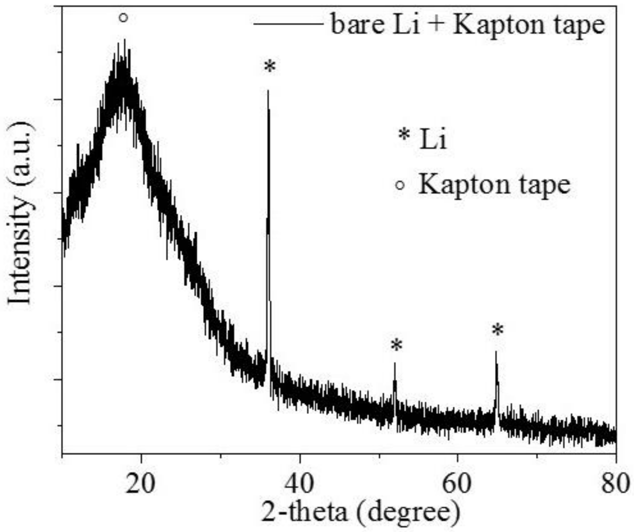

Supplementary Figure 1 XRD characterization of bare Li electrode.

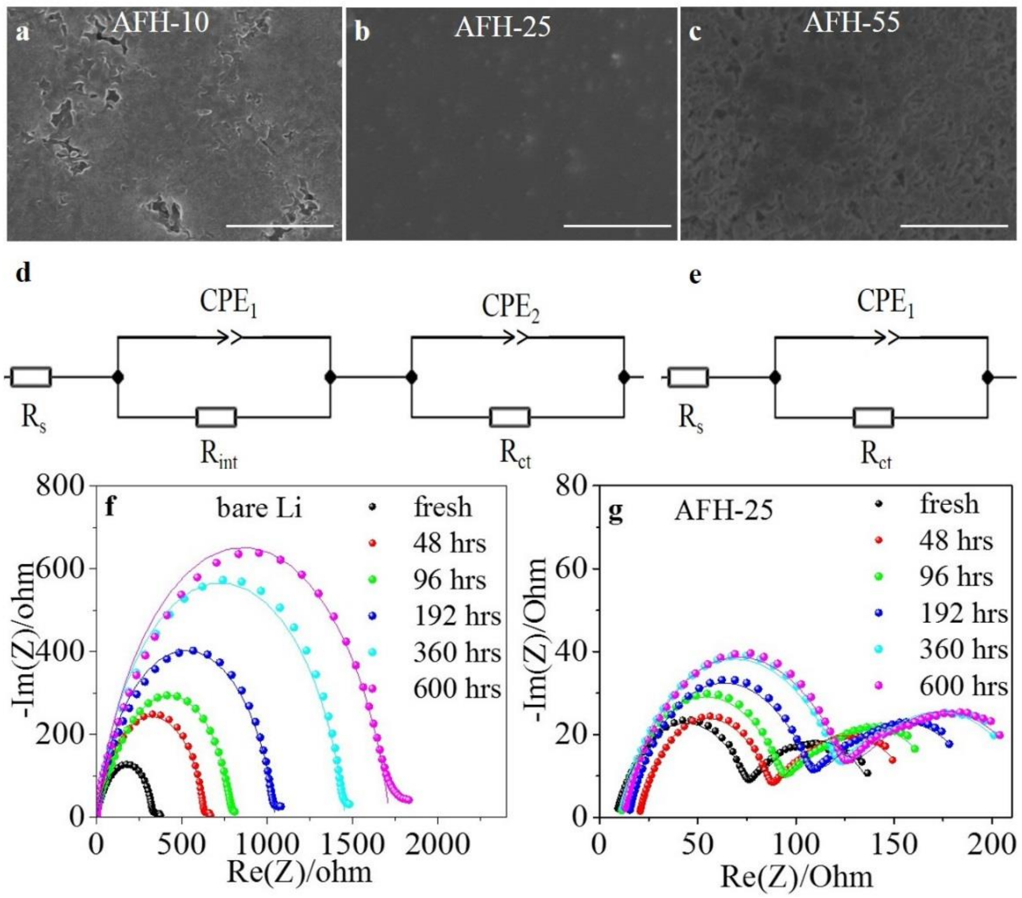

Supplementary Figure 2 Morphology characterization and stability test. **a-c** SEM image of plated Li on AFH-10, AFH-25, and AFH-55 after 1st plating at 0.5 mA cm^{-2}, respectively. The scale bars are 20μm. **d, e** The equivalent circuit for fitting Nyquist plot with a double and single semicircle, respectively. **f, g** Nyquist plot of bare Li and AFH-25 symmetrical cells as a function of hrs, respectively.

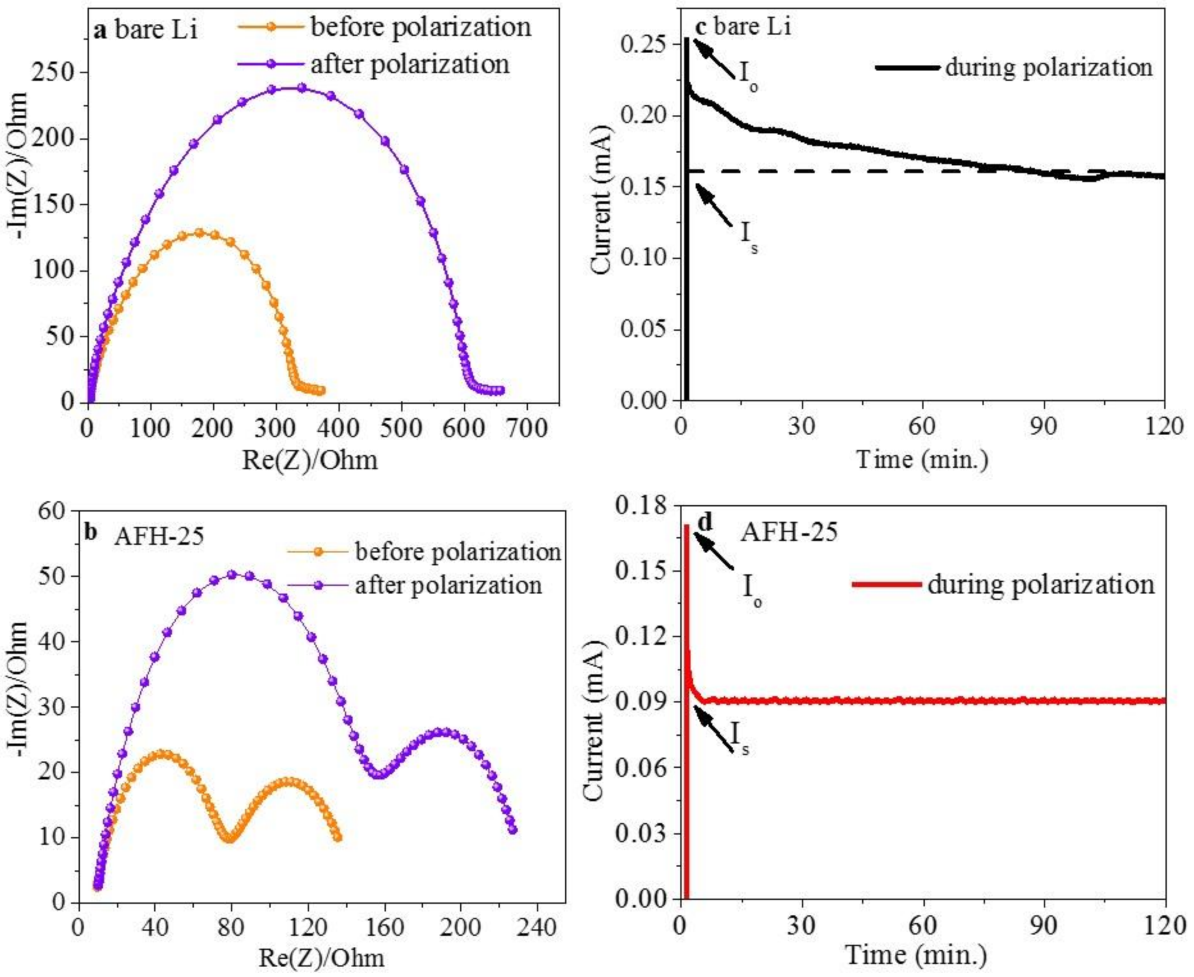

Supplementary Figure 3 Li-ion transference number measurement. **a, b** Nyquist plots before and after polarization in bare Li and AFH-25 symmetrical cell, respectively. **c, d** The polarization curve of bare Li and AFH-25 symmetrical cell, respectively using chronoamperometry.

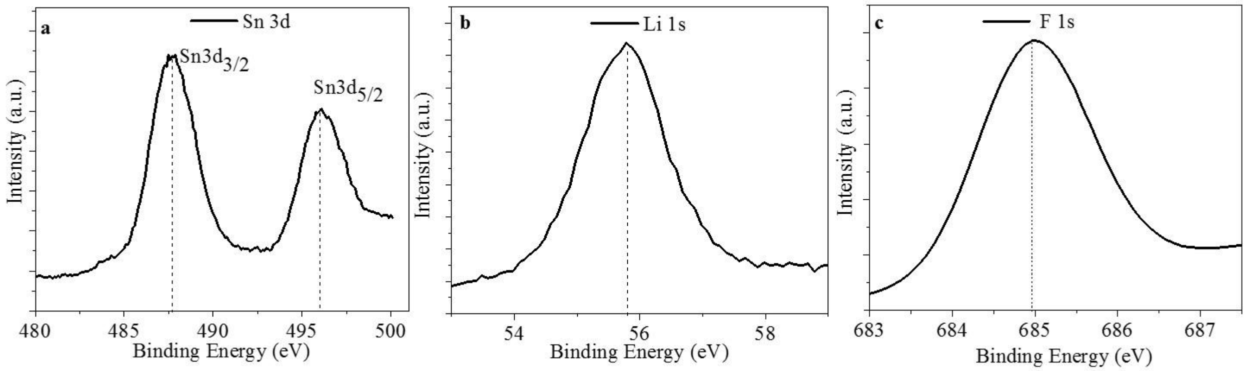

Supplementary Figure 4 XPS analysis of AFH-25. The core-level of (**a**) Sn 3d, (**b**) Li 1s and (**c**) F 1s.

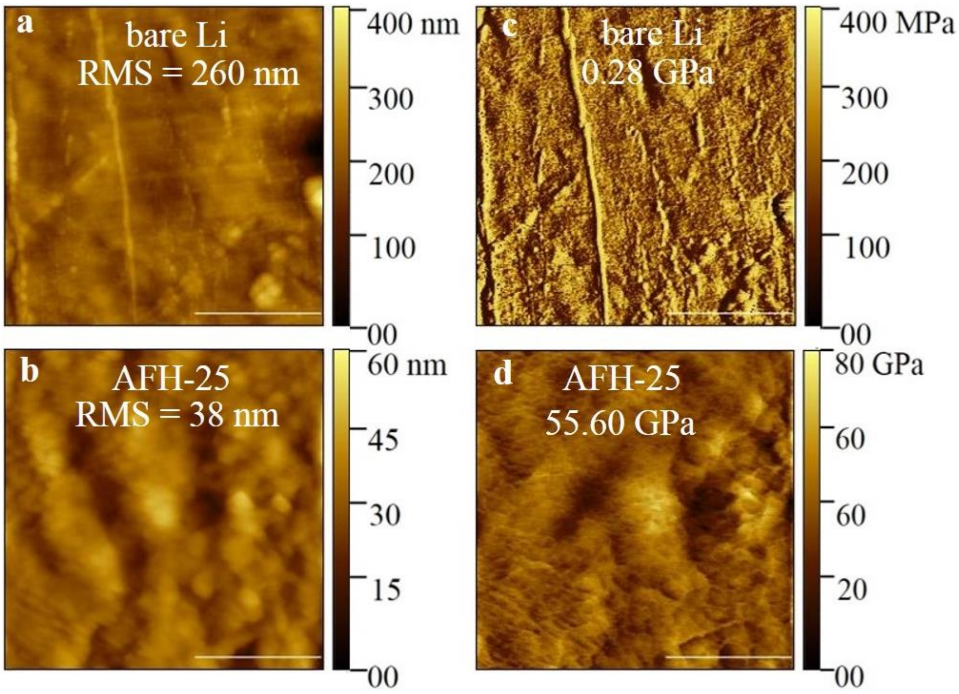

Supplementary Figure 5 AFM surface topography and Young's modulus analysis. a, b The surface topography of bare Li and AFH-25, respectively. **c, d** The corresponding Young's modulus mapping from **a** and **b**, respectively. The scale bars are 4μm.

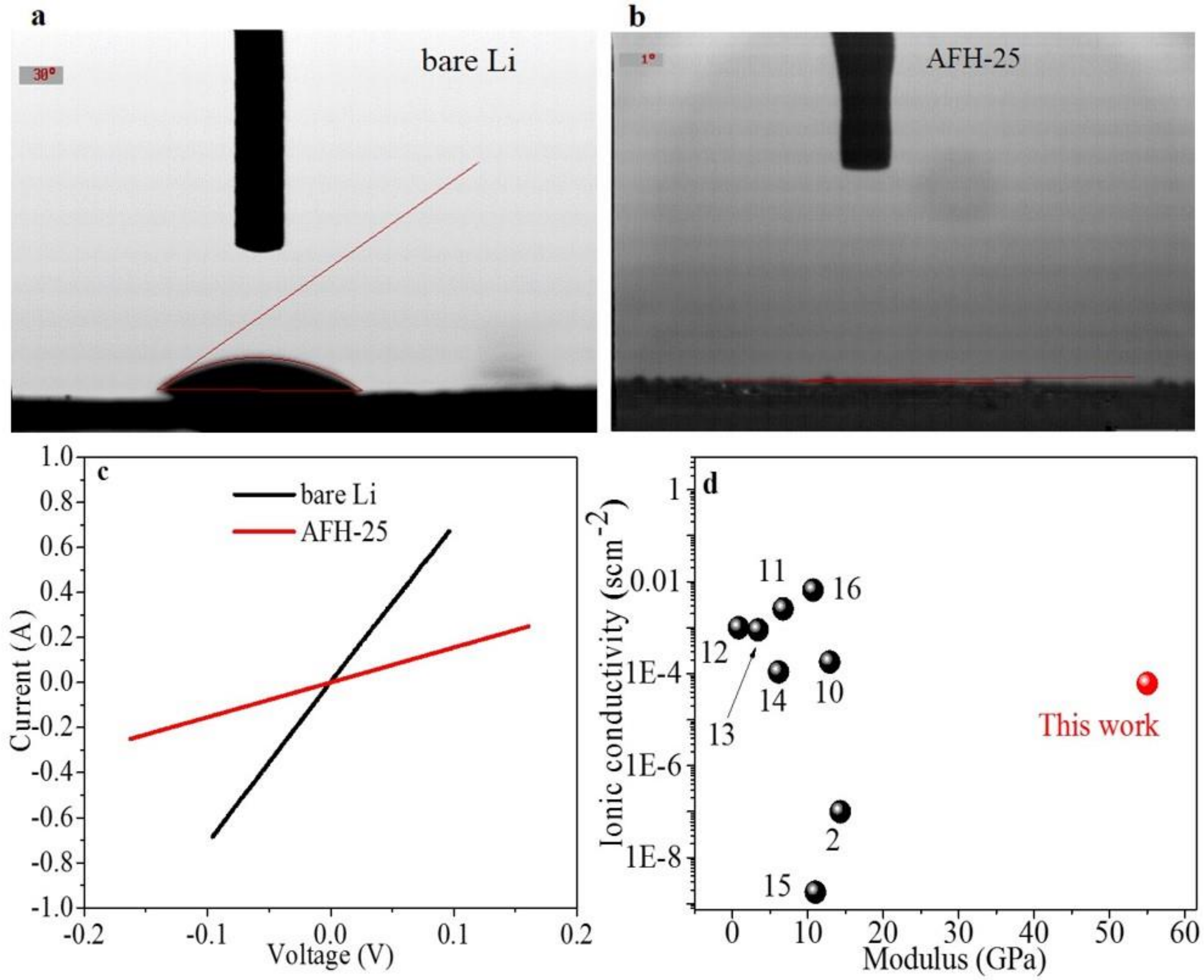

Supplementary Figure 6 Wettability and conductivity measurement. **a, b** The contact angle measurement of bare Li and AFH-25, respectively. **c** LSV measurement of bare Li and AFH-25. **d** The comparison of Young's modulus and ionic conductivity value of artificial SEI with previous reports.

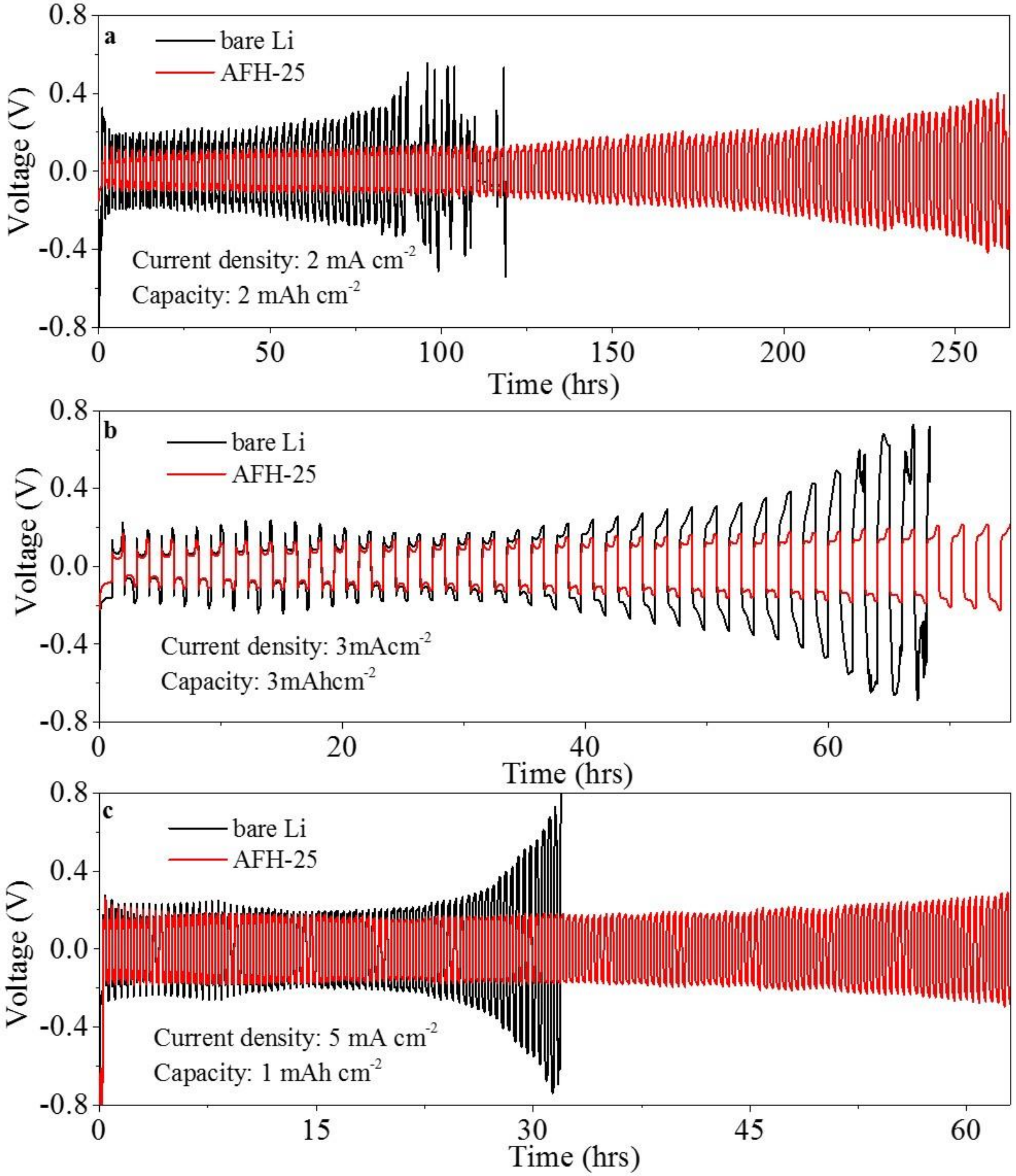

Supplementary Figure 7 Electrochemical symmetric cell test. a-c Voltage versus time profile of bare and AFH-25 symmetrical cells at different current density and capacity.

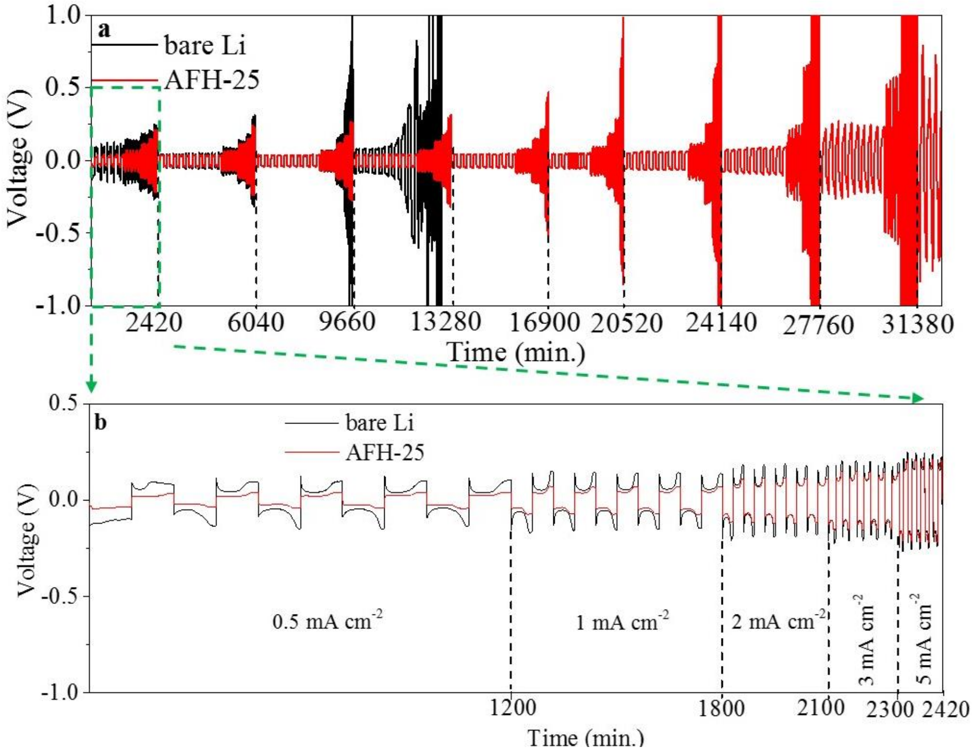

Supplementary Figure 8 Electrochemical symmetric cell test. a Voltage versus time profile of bare and AFH-25 symmetrical cells at different current density with a fixed capacity of 1 mAh cm^{-2}. **b** Zoom-in voltage versus time profile in the initial plating/stripping time from **a**.

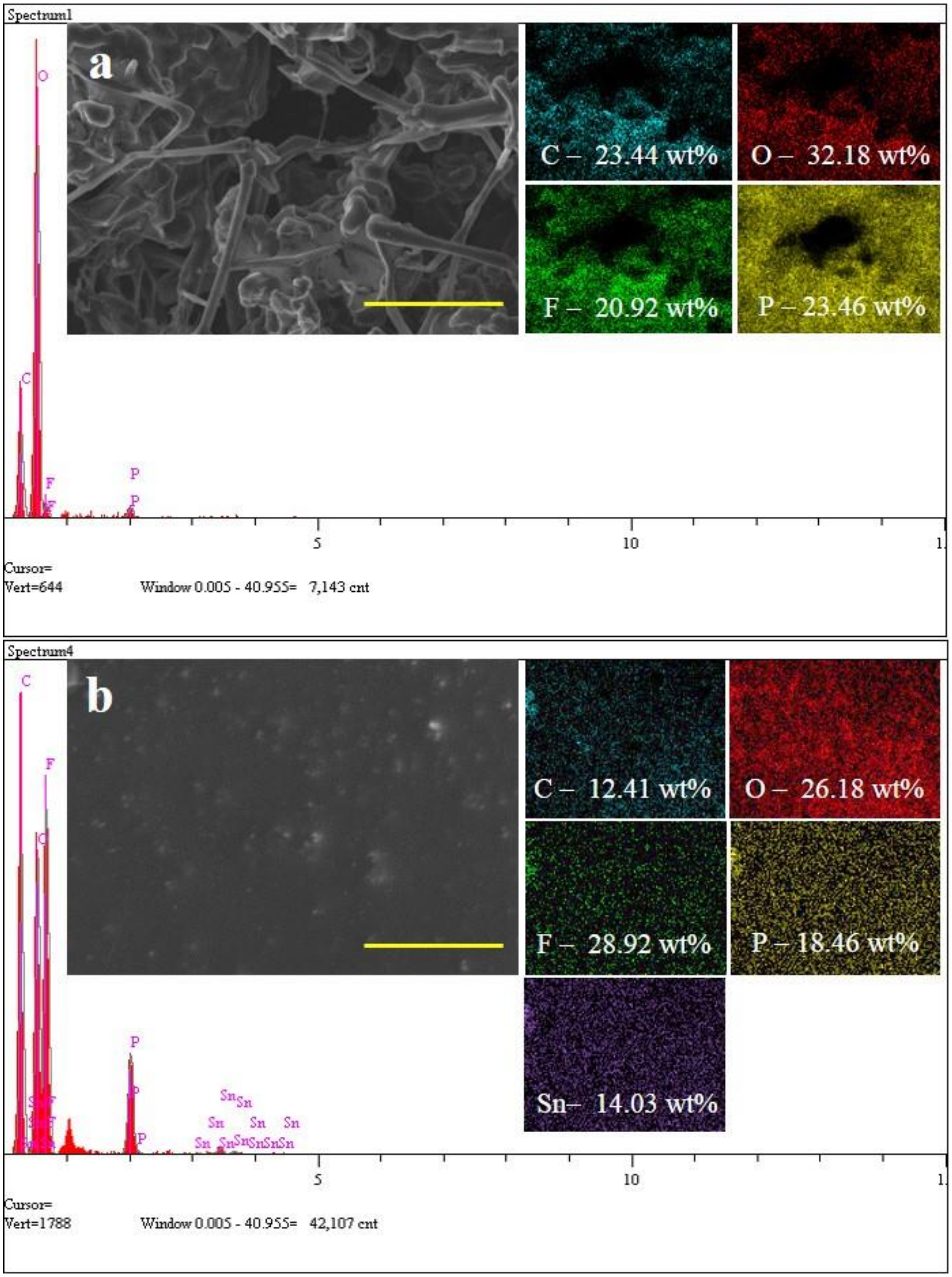

Supplementary Figure 9 SEM imaging and EDS elemental mapping. a, b The top-view SEM images, corresponding EDS and elemental mapping of the aforementioned area of bare Li and AFH-25 after 1st plating, respectively. The scale bars are 20 μm.

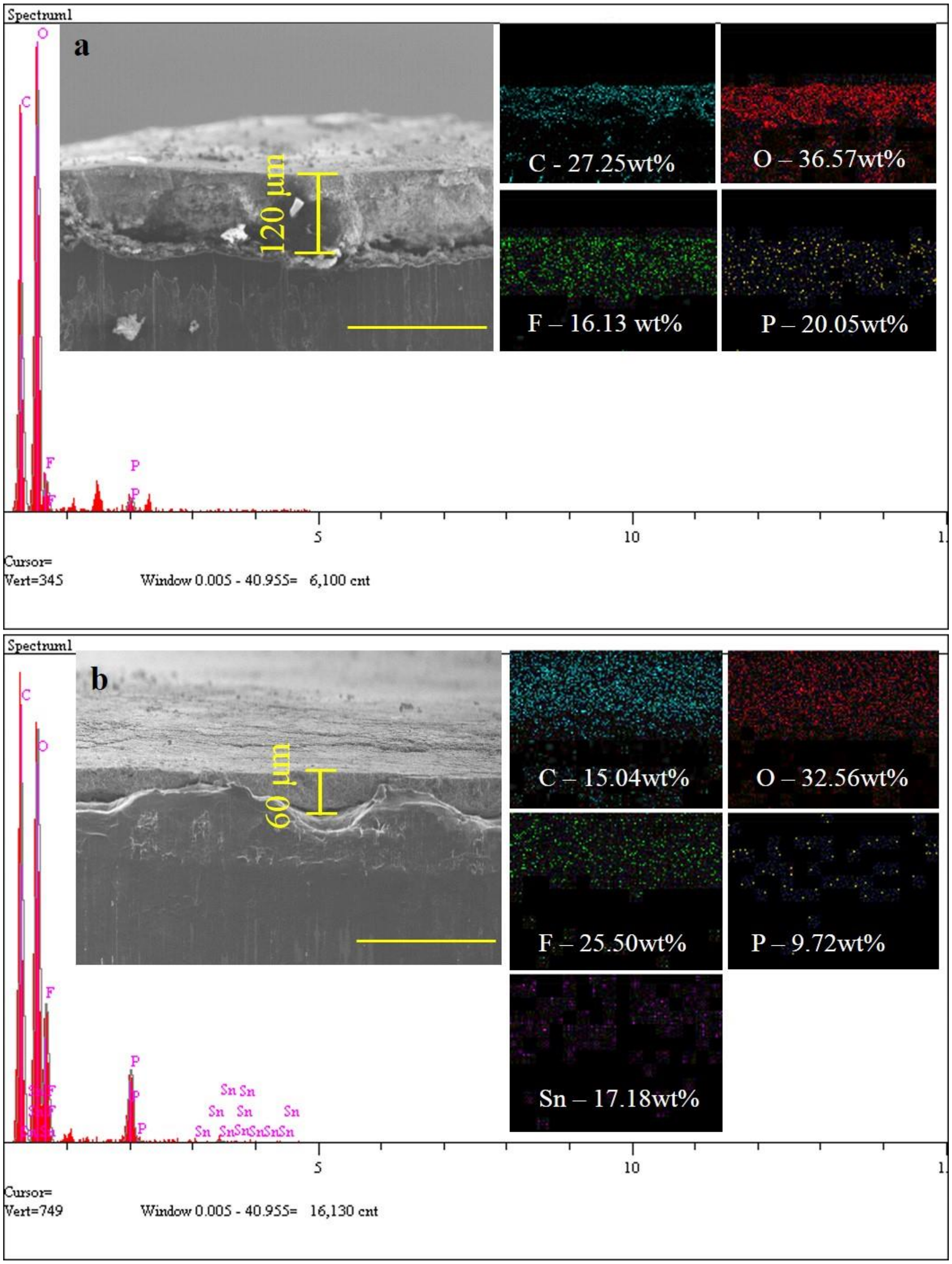

Supplementary Figure 10 SEM imaging and EDS elemental mapping. a, b The cross-sectional SEM images, corresponding EDS and elemental mapping of the aforementioned area. of bare Li and AFH-25 after 100th plating, respectively. The scale bars are 200 μm.

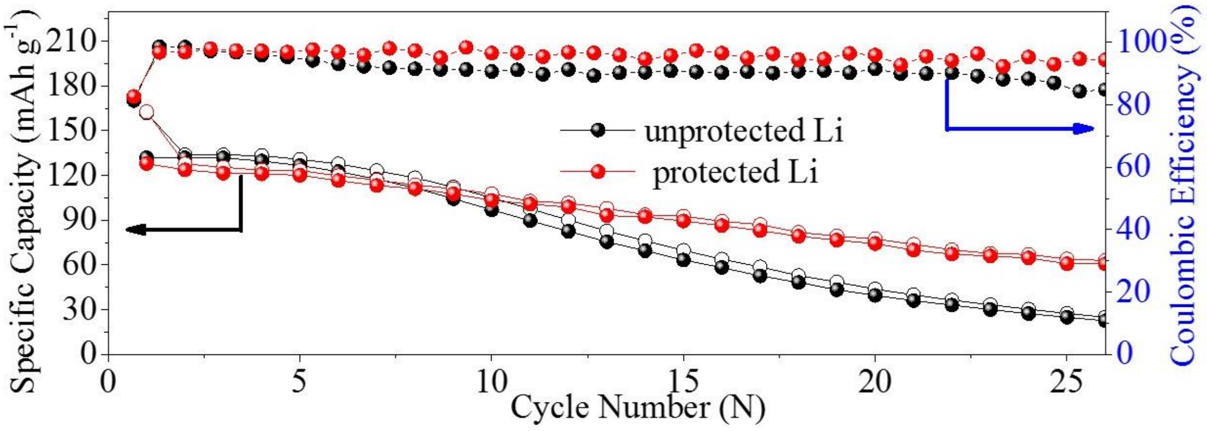

Supplementary Figure 11 The electrochemical performance of NMC11/Li. The cycling performance test is at the current density of 1C with N/P ratio = 2:1.

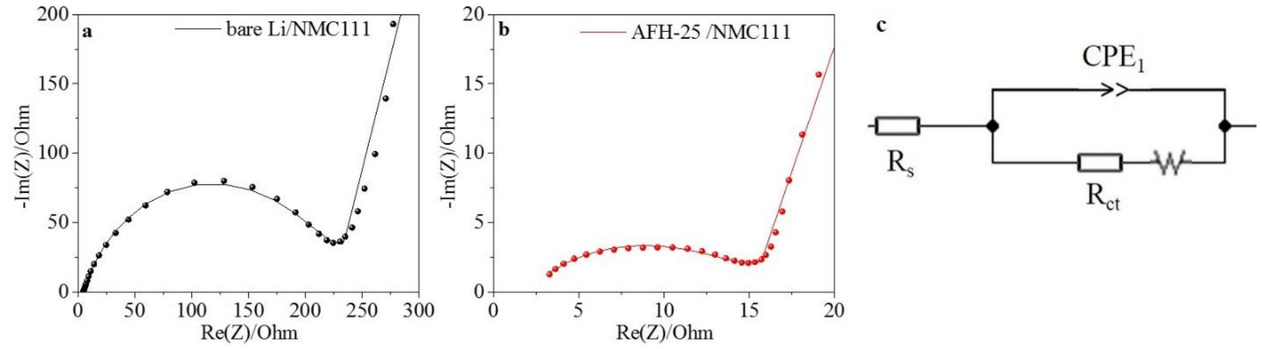

Supplementary Figure 12 EIS measurement of full cells. a bare Li as an anode. **b** AFH-25 as an anode. **c** The equivalent circuit used for fitting the EIS results of **a** and **b**.

Supplementary Tables

Supplementary Table 1 Impedance results of bare Li, and AFH-10, AFH-25 and AFH-55 Li symmetric cells.

	R_s (Ω)	R_{int} (Ω)	R_{ct} (Ω)
Bare Li	2.39	-	374.50
AFH-10	9.27	120.60	77.36
AFH-25	9.59	79.14	47.00
AFH-55	10.53	114.80	69.10

Supplementary Table 2 Impedance results of symmetric cells with bare Li and AFH-25 as a function of hours.

Time (hrs)	Bare Li			AFH-25		
	$R_s(\Omega)$	R_{int} (Ω)	R_{ct} (Ω)	$R_s(\Omega)$	R_{int} (Ω)	R_{ct} (Ω)
fresh	2.01	-	343.50	9.59	79.14	47.00
48	2.39	-	638.60	9.22	68.14	67.75
96	2.23	-	797.90	10.64	84.68	68.48
192	2.08	-	979.00	11.36	95.89	67.91
360	2.12	-	1508.00	10.72	168.5	106.8
600	2.52	-	1717.00	11.90	115.6	75.75

Supplementary Table 3 The comparison of plating/stripping hours of this work with previous reports.

Ref.	artificial layer type		Electrolyte	density/ capacity (mA cm^{-2}/ mAh cm^{-2})	Plating/ stripping (hours)
1	Al_4Li_9-LiF nanoparticles as the ideal skeleton for Li metal		1 M $LiPF_6$ EC/DEC (1:1, v/v) 10% FEC+1% VC	1 /1	~195
				10/1	~195
				20/1	~195
2	Al_2O_3 thin film sputtered on Li		1 M $LiPF_6$ EC/DEC/DMC (1:1:1, v/v/v)	0.5/0.5	~1200
3	LiF host constructed on Li metal by surface reaction with NH_4HF_2		1 M LiTFSI DOL/DME (1:1, v/v)	1/1	~550
				2/1	~400
				5/1	~390
4	Mo_6S_8/Carbon coated Li		1 M $LiPF_6$ EC/DMC (1:1, v/v)	1/1	~600
5	Lithiated-10 nm MoS_2 sputtered Li		1 M LiTFSI DOL/DME (1:1,v/v)	1/1	~265
6	Coating cyclic ether group-containing polymer on Li surface		1M $LiPF_6$ EC/EMC/FEC (3:7:1, v/v)	0.5/1	~300
7	Surface fluorination of Li using fluoropolymer		1M $LiPF_6$ EC/DEC (1:1, v/v)	1/1	~ 600
				3/1	~120
				5/1	~120
8	Pretreat Li with SnTFSI containing electrolyte		1M $LiPF_6$ EC/DMC (1:1, v/v)+ 10v% FEC	3/1	~500
9	In situ formed films comprised of lithium-based alloys	$Li_{13}In_3$	1M LiTFSI DOL/DME (1:1, v/v)	2/2	~1250
		LiZn			~1050
		Li_3Bi			~1450
		Li_3As			~1200
		LiZn	1M $LiPF_6$ EC/DMC		~400
		Li_3As			~325
		$Li_{13}In_3$			~275
This work	Artificial fluorinated hybrid SEI on Li electrode using SnF_2		1M $LiPF_6$ EC/DEC (1:1)	0.5/1	~2325
				1/1	~850
				2/2	~265
				3/3	~75
				5/1	~65

Supplementary Table 4. Capacities of the bare Li/NMC111 and AFH-25 /NMC111 at different current densities.

Rates	Cycle no.	Bare Li	AFH-25
		Charge/discharge capacity (mAh g^{-1})	Charge/discharge capacity (mAh g^{-1})
0.1C	3	150.86/147.18	151.38/148.22
0.2C	8	145.75/144.28	147.28/145.78
1C	13	124.55/122.77	130.14/129.58
3C	18	102.57/202.60	115.50/115.09
5C	23	85.29/84.60	100.54/100.44
0.1C	28	147.86/144.71	151.10/147.25

Supplementary Table 5 Impedance results for Li/NMC111 cells using bare Li and AFH-25 as an anode.

	R_s (Ω)	R_{ct} (Ω)
Bare Li/NMC111	4.79	189.00
AFH-25/NMC111	2.74	12.91

Supplementary Methods

Lithium-ion (Li^+) transference number ($T_{Li}{}^+$) (**Supplementary Figure 3)** was calculated using Bruce–Vincent–Evans equation $T_{Li}{}^+ = Is.(\Delta V - IoRo)/(Io(\Delta V - IsRs)$, where ΔV is the applied polarization voltage, I_o and R_o are the initial currents and initial resistance(charge transfer resistance) before polarization, I_s and R_s are steady-state current and steady-state resistance (sum of interfacial resistance and charge transfer resistance) after polarizations for 7200 seconds, respectively.

Linear sweep voltammetry (LSV) was performed at 100 mV s^{-1} within a voltage range of −0.2 to 0.2 as shown in **Supplementary Figure 6**c. Both the bare Li and AFH-25 were separately sandwiched between stainless steel spacer and crimped in the battery casing inside the Ar-glove box to avoid moisture contact.

Supplementary Figure 6d shows that an artificial SEI in this work has fairly high enough ionic conductivity sufficient to diffuse the Li-ions and large enough Young modulus to suppress the Li dendrite growth [2, 10, 11, 12, 13, 14, 15, 16]. The method of calculating ionic conductivity of an artificial SEI was based on EIS measurement of symmetrical cells assembled with bare Li and AFH-25 respectively as presented by Cui *et al.*[17] Ionic conductivity of the protective layer can be calculated by using an equation $\sigma = \frac{2L}{(R.a)}$, where L is the thickness, 'R' is the resistance and 'a' is the area of SEI the layer.

To study the full cell performances (**Supplementary Figure 11**) with N/P =2 ratio, the predetermined Li anode capacity was electroplated on stainless steel as an electrode using 1M $LiPF_6$ in EC/DEC followed by rinsing with DMC. The Li electrodeposited stainless steel was designated as unprotected Li and electrodeposited Li treated with SnF_2 was designated as

protected Li. Again, the full cells with NMC111 cathode coupled with unprotected and protected Li were cycled using 1M $LiPF_6$ EC: DEC as an electrolyte.

Supplementary References

1. Wang, H., Lin, D., Liu, Y., Li, Y. & Cui, Y. Ultrahigh-current density anodes with interconnected Li metal reservoir through overlithiation of mesoporous AlF_3 framework. *Sci. Adv.* **3**, e1701301 (2017).

2. Wang, L. et al. Long lifespan lithium metal anodes enabled by Al_2O_3 sputter coating. *Energy Storage Mater.* **10**, 16–23 (2018).

3. Yuan, Y. et al. Regulating Li deposition by constructing LiF-rich host for dendrite-free lithium metal anode. *Energy Storage Mater.* **16**, 411–418 (2019).

4. Lu, K., Gao, S., Dick, R. J., Sattar, Z. & Cheng, Y. A fast and stable Li metal anode incorporating an Mo_6S_8 artificial interphase with super Li-ion conductivity. *J. Mater. Chem. A* **7**, 6038–6044 (2019).

5. Cha, E. et al. 2D MoS_2 as an efficient protective layer for lithium metal anodes in high-performance Li-S batteries. *Nat. Nanotechnol.* **13**, 521–521 (2018).

6. Gao, Y. et al. Interfacial chemistry regulation via a skin-grafting strategy enables high-performance lithium-metal batteries. *J. Am. Chem. Soc.* **139**, 15288–15291 (2017).

7. Zhao, J. et al. Surface fluorination of reactive battery anode materials for enhanced stability. *J. Am. Chem. Soc.* **139**, 11550–11558 (2017).

8. Tu, Z. et al. Fast ion transport at solid–solid interfaces in hybrid battery anodes. *Nat. Energy* **3**, 310 (2018).

9. Liang, X. et al. A facile surface chemistry route to a stabilized lithium metal anode. *Nat. Energy* **2**, 17119 (2017).

10. Yan, C. et al. An armored mixed conductor interphase on a dendrite-free lithium-metal anode. *Adv. Mater.* 30, 1804461 (2018).

11. Xu, R. et al. Artificial soft-rigid protective layer for dendrite-free lithium metal anode. *Adv. Funct. Mater.* **28**, 1705838 (2018).

12. Liu, Y. et al. An artificial solid electrolyte interphase with high Li-ion conductivity, mechanical strength, and flexibility for stable lithium metal anodes. *Adv. Mater.* **29**, 1605531 (2017).

13. Lee, H., Lee, D. J., Kim, Y. J., Park, J. K. & Kim, H. T. A simple composite protective layer coating that enhances the cycling stability of lithium metal batteries. *J. Power Source* **284**, 103–108 (2015).

14. Li, S., Fan, L. & Lu, Y. Rational design of robust-flexible protective layer for safe lithium metal battery. *Energy Storage Mater.* **18**, 205–212 (2019).

15. Li, N. W., Yin, Y. X., Yang, C. P. & Guo, Y. G. An artificial solid electrolyte interphase layer for stable lithium metal anodes. *Adv. Mater.* **28**, 1853–1858 (2016).

16. Wang, M. et al. Effect of LiFSI concentrations to form thickness-and modulus-controlled SEI layers on lithium metal anodes. *J. Phys. Chem. C* **122**, 9825–9834 (2018).

17. Zhao, J. et al. Air-stable and freestanding lithium alloy/graphene foil as an alternative to lithium metal anodes. *Nat. Nanotechnol.* **12**, 993 (2017).
